# SPLUNC1 is a negative regulator of the Orai1 Ca^2+^ channel

**DOI:** 10.14814/phy2.15306

**Published:** 2022-05-17

**Authors:** Tongde Wu, Alexandra S. Goriounova, Erin N. Worthington, Joe A. Wrennall, Arunava Ghosh, Saira Ahmad, M. Flori Sassano, Robert Tarran

**Affiliations:** ^1^ Department of Cell Biology & Physiology The University of North Carolina at Chapel Hill North Carolina 27599 USA; ^2^ Department of Pharmacology The University of North Carolina at Chapel Hill North Carolina 27599 USA; ^3^ Divison of Pulmonology Department of Pediatrics The University of North Carolina at Chapel Hill North Carolina 27599 USA; ^4^ Division of Pulmonology, Department of Pediatrics Carilion Clinic and Virginia Tech Carilion School of Medicine Roanoke Virginia 24016 USA

**Keywords:** airway, airway smooth muscle, BPIFA1, calcium, FRET, NEDD4‐2

## Abstract

Orai1 is a ubiquitously‐expressed plasma membrane Ca^2+^ channel that is involved in store‐operated Ca^2+^ entry (SOCE): a fundamental biological process that regulates gene expression, the onset of inflammation, secretion, and the contraction of airway smooth muscle (ASM). During SOCE, Ca^2+^ leaves the endoplasmic reticulum, which then stimulates a second, amplifying wave of Ca^2+^ influx through Orai1 into the cytoplasm. Short Palate LUng and Nasal epithelial Clone 1 (SPLUNC1; gene name BPIFA1) is a multi‐functional, innate defense protein that is highly abundant in the lung. We have previously reported that SPLUNC1 was secreted from epithelia, where it bound to and inhibited Orai1, leading to reduced SOCE and ASM relaxation. However, the underlying mechanism of action is unknown. Here, we probed the SPLUNC1‐Orai1 interactions in ASM and HEK293T cells using biochemical and imaging techniques. We observed that SPLUNC1 caused a conformational change in Orai1, as measured using Forster resonance energy transfer (FRET). SPLUNC1 binding also led to Nedd4‐2 dependent ubiquitination of Orai1. Moreover, SPLUNC1 internalized Orai1 to lysosomes, leading to Orai1 degradation. Thus, we conclude that SPLUNC1 is an allosteric regulator of Orai1. Our data indicate that SPLUNC1‐mediated Orai1 inhibition could be utilized as a therapeutic strategy to reduce SOCE.

## Graphical abstract

Orai1 is a ubiquitously‐expressed plasma membrane Ca^2+^ channel that is involved in store‐operated Ca^2+^ entry (SOCE): A fundamental biological process that regulates gene expression, the onset of inflammation, secretion, and the contraction of airway smooth muscle (ASM). We have previously found that Short Palate LUng and Nasal epithelial Clone 1 (SPLUNC1; gene name BPIFA1) is secreted from epithelia where it can bind to and inhibit Orai1, leading to reduced SOCE and ASM relaxation. Here, we probed the SPLUNC1‐Orai1 interactions in ASM and HEK293T cells, and found that SPLUNC1 causes allosteric changes in Orai1 upon binding that lead to Orai1 ubiquitination, internalization, and degradation.

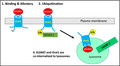

## 1 INTRODUCTION

Ca^2+^ is an important, universal intracellular second messenger, and appropriate Ca^2+^ homeostasis is essential for every mammalian cell. Store‐operated Ca^2+^ entry (SOCE) is a common mechanism for Ca^2+^ regulation (Putney, 1986). The Ca^2+^ release‐activated Ca^2+^ (CRAC) channel is the prototypical SOCE channel (Prakriya & Lewis, 2015). The identification of stromal interaction molecule 1 (STIM1) as the endoplasmic reticulum Ca^2+^ sensing protein, and Orai1 as the pore‐forming subunit of the CRAC were breakthroughs that triggered a wave of advances in elucidating the molecular mechanisms and functions of CRAC in many cells and tissues (Hogan & Rao, 2015). The binding of inositol 1,4,5 trisphosphate (IP_3_) to the IP_3_ receptor (IP_3_R) on the endoplasmic reticulum membrane triggers release of endoplasmic reticulum Ca^2+^ stores. Reductions in endoplasmic reticulum Ca^2+^ levels are sensed by STIM1 via its EF‐hand Ca^2+^ binding motif, which triggers STIM1 to translocate to the junctions of the endoplasmic reticulum and plasma membrane, where STIM1 interacts with Orai1 and induces Ca^2+^ entry. Increases in cytoplasmic Ca^2+^ exert a broad range of physiological effects including activation of the Ca^2+^‐calcineurin‐NFAT (nuclear factor of activated T cells) pathway, cell proliferation, and cell death depending on the amplitude or duration of Ca^2+^ and the binding proteins (Hewavitharana et al., 2008; Shaw et al., 2013; Srikanth & Gwack, 2012). In ASM, increases in cytosolic Ca^2+^ lead to myosin light chain phosphorylation and cause muscle contraction. Orai1 is widely expressed throughout the body (Gwack et al., 2007), and a number of disease‐causing mutations have been described (Lacruz & Feske, 2015): Patients with global loss‐of‐function Orai1 mutations have severe combined immune deficiency (SCID) syndrome. In contrast, gain‐of‐function Orai1 mutations cause tubular aggregate myopathy (TAM), York platelet syndrome, and Stormorken syndrome (Feske et al., 2006).

The Orai1 protein contains four alpha‐helical transmembrane domains (TM1–TM4), two extracellular loops, one intracellular loop, as well as cytoplasmic N‐ and C‐termini that mediate the interaction with STIM1, and other regulatory proteins (Shaw et al., 2013; Srikanth & Gwack, 2012). Functional CRAC channels are composed of hexameric Orai1 proteins, with six TM1 domains, one from each Orai1 subunit, forming the central ion pore. TM1 contains several amino acid residues that define the biophysical properties of the channel. Mutations of these amino acids either make the pore non‐selective or block ion conduction (Hou et al., 2012). Orai1 can be regulated by post‐translational modifications. For instance, Orai1 phosphorylation at serine (S) S27 and S30 in the cytoplasmic N terminus suppressed SOCE (Kawasaki et al., 2010). However, little is known about Orai1 trafficking.

Short palate and nasal epithelial clone 1 (SPLUNC1), also known as Bacterial Permeability Increasing family member A1 (BPIFA1), Secretory Protein in Upper Respiratory Tract (SPURT), Lung‐specific X protein (LUNX) and Nasopharyngeal Carcinoma‐Related Protein, is a multifunctional protein that is secreted by airway epithelia and other organs (Tarran & Redinbo, 2014). Its N‐terminus regulates the epithelial Na^+^ channel (ENaC) to modulate salt/water balance and airway hydration, while the central body of SPLUNC1 has surfactant‐like properties and has antimicrobial activity against Gram‐negative bacteria (Di, 2011; Gakhar et al., 2010; Garcia‐Caballero et al., 2009; McGillivary & Bakaletz, 2010; Walton et al., 2016). We previously demonstrated that SPLUNC1 was secreted bilaterally by airway epithelia and that SPLUNC1’s C‐terminus inhibited Orai1‐dependent Ca^2+^ influx (Wu et al., 2017). Moreover, SPLUNC1 expression was diminished in asthmatic airways leading to greater Ca^2+^ signaling. Consistent with these observations, SPLUNC1^−/−^ mice exhibited spontaneous ASM contraction and airway hyper‐reactivity, which could be corrected by the addition of recombinant SPLUNC1 (Wu et al., 2017). Despite the body of evidence demonstrating that SPLUNC1 negatively regulates Orai1, the underlying mechanism of action remains obscure. Here, we explored how SPLUNC1 negatively regulates Orai1 protein levels in ASM and HEK293T cells.

### 1.1 METHODS AND MATERIALS

### 1.2 DNA constructs

Yellow fluorescent protein (YFP) and Cyan fluorescent protein (CFP)‐tagged human Orai1 were kind gifts from Dr. Jim Putney at the National Institute of Environmental Health Science (NIEHS). pcDNA3.1(+)‐V5‐SPLUNC1 was generously provided by Dr. Colin Bingle at the University of Sheffield. Myc‐tagged Orai1 and HA‐tagged Nedd4‐2 were commercially purchased (Addgene plasmid #12199 and #27000). Nedd4‐2‐C821A (Nedd4‐2 (DN)) was a kind gift offered by Dr. Pete Snyder (University of Iowa). tdTomato‐2xOrai1, tdTomato‐4xOrai1, and tdTomato‐6xOrai1 constructs were kind gifts from Dr. Donald Gill at the Pennsylvania State University College of Medicine.

### 1.3 Cell culture and transfection

ASM cells were maintained in Dulbecco's Modified Eagle Medium: Nutrient Mixture F‐12 (DMEM/F12; Thermo Fisher) supplemented with 10% fetal bovine serum (FBS; Sigma Aldrich) and 0.1% penicillin‐streptomycin (Thermo Fisher). HEK293T cells were purchased from ATCC (catalogue number: CRL‐3216) and maintained in DMEM (Thermo Fisher) in the presence of 10% FBS and 0.1% penicillin–streptomycin. DNA transfections were performed using Lipofectamine 2000 reagent (Thermo Fisher) according to the manufacturer's instructions.

### 1.4 Immunoprecipitation and immunoblot analysis

The following commercially available antibodies were used: rabbit anti‐Orai1 (1:1000), anti‐HA epitope (1:2000) and anti‐myc epitope (1:1000) (Santa Cruz), anti‐GAPDH (glyceraldehyde‐3‐phosphate dehydrogenase, 1:3000) (Cell Signaling Technology); mouse anti‐V5 epitope (1:2000) (Thermo Fisher) and anti‐FLAG epitope (1:2000) (Sigma Aldrich); goat anti‐SPLUNC1 (1:2000) (R&D Systems); peroxidase‐conjugated donkey anti‐mouse IgG (H + L), peroxidase‐conjugated donkey anti‐rabbit IgG (H + L), peroxidase‐conjugated donkey anti‐goat IgG (H+L) (all 1:3000, Jackson ImmunoResearch). To detect protein expression in total cell lysates, cells were lysed in Pierce IP lysis buffer (25 mM Tris‐HCl pH 7.4, 150 mM NaCl, 1% NP‐40, 1 mM EDTA, 5% glycerol), supplemented with 1 × proteinase inhibitor cocktail (Roche), followed by sodium dodecyl sulfate (SDS)–polyacrylamide electrophoresis and immunoblot. For immunoprecipitation, cells were lysed 48 h post‐transfection in Pierce IP lysis buffer plus protease inhibitors. Cell lysates were pre‐cleared with protein A/G agarose beads and then incubated with 1 μg of antibodies against either the V5‐ or Myc‐epitope with protein A/G agarose beads on a rotator at 4 °C overnight. After three washes with IP lysis buffer, immunoprecipitated complexes were eluted in sample buffer (50 mM Tris‐HCl (pH 6.8), 2% SDS, 10% glycerol, 5% (v/v) β‐mercaptoethanol (BME), 0.1% bromophenol blue) by heating the samples at 95 °C for 5 min, followed by SDS–polyacrylamide gel electrophoresis, and then subjected to immunoblot analysis.

### 1.5 Expression and purification of human SPLUNC1

cDNA of SPLUNC1 lacking its N‐terminal signal secretion sequence (i.e., the first 19 amino acids) was transformed into BL21‐Codon Plus competent cells (Agilent Technologies) and bacterially expressed protein was purified as previously described (Garland et al., 2013).

### 1.6 Protein half‐life measurements

To measure the half‐life of Orai1, 50 µM cycloheximide (CHX, Sigma) was added to block protein synthesis. Total cell lysates were collected at different time points and immunoblotted with anti‐Orai1 and anti‐tubulin antibodies. Band intensity was quantified using the ChemiDoc MP gel documentation system and Image Lab 5.0 software (Bio‐Rad).

### 1.7 mRNA extraction and quantitative real‐time PCR (qRT‐PCR)

Total mRNA was extracted from cells using RNaEasy reagent (Qiagen). Equal amounts of RNA (1 µg) were used for reverse transcription using a cDNA synthesis kit (BioRad). The following TaqMan probes were used: human Orai1 (assay ID: Hs03046013_m1) and human GAPDH (assay ID: Hs02786624_g1) (Thermo Fisher). qRT‐PCR was performed on the Applied Biosystems 7500 fast machine (Thermo Fisher) as follows: one cycle of initial denaturation (95 C for 4 min), 45 cycles of amplification (95 C for 10s and 60 C for 30s). Data were normalized to GAPDH.

### 1.8 Ubiquitylation of Orai1

HEK293T cells transfected with expression plasmids (for the indicated proteins) for 48 h were treated for 16 h with 100 µM chloroquine (Sigma) to block protein degradation. Cells were then lysed in a buffer containing 2% SDS, 150 mM NaCl, 10 mM Tris‐HCl, and 1 mM dithiothreitol (DTT). The cell lysates were boiled immediately for 10 min to inactivate cellular ubiquitin hydrolases and preserve ubiquitin–protein conjugates. The boiled lysates were then cooled and diluted five‐fold with a Tris‐buffered salt (TBS) solution (without SDS) and used for immunoprecipitation with an antibody against the Myc epitope. Immunoprecipitated proteins were immunoblotted with an antibody against HA‐ or Flag‐tag.

### 1.9 Confocal microscopy and acceptor photobleaching Forster Resonance Energy Transfer (FRET)

All images were taken on a Leica TCS SP8 confocal microscope, using the Leica Application Suite X software (Leica) with a 63 × 1.4 NA oil immersion objective lens. For FRET, cells were co‐transfected with YPF‐Orai1 and CFP‐Orai1 for 24 h. Following treatment with purified SPLUNC1 (10 µM), cells were fixed with 4% PFA in PBS for 10 min at room temperature. Acceptor photobleaching FRET was performed as previously described (Staruschenko et al., 2004). Briefly, the donor (CFP) was excited at 405 nm and emission collected from 470 to 525 nm. The acceptor (YFP) was excited at 485 nm and emission collected from 550 to 600 nm. The % FRET efficiency was calculated as $\left[\frac{Donor^{Post‐bleach}‐Donor^{pre‐bleach}}{Donor^{Post‐bleach}}\right]\times 100.$ Three measurements were taken per cell. We used simultaneously‐acquired, transmitted light images to identify cell boundaries. Using these images, regions of interest were placed around distinct areas of the plasma membrane. Any observable sub‐membrane vesicles or puncta were excluded from the analysis.

### 1.10 Data analysis

All images and western blots were analyzed using Image J (NIH Freeware), data were processed in Microsoft Excel and all graphs were plotted in GraphPad Prism. Statistical analyses were performed using either GraphPad Prism or GraphPad Instat. All data were presented as the mean ± SEM. for n experiments. Data were analyzed using ANOVA or Student's *t*‐test, the Kruskal–Wallis test with Dunn's multiple comparisons test or the Mann‐Whitney test as appropriate. Differences between groups were assessed using analysis of variance. From such comparisons, differences yielding *p* ≤ .05 were judged to be significant.

## 2 RESULTS

### 2.1 SPLUNC1 induces Orai1 degradation

**FIGURE 1:**
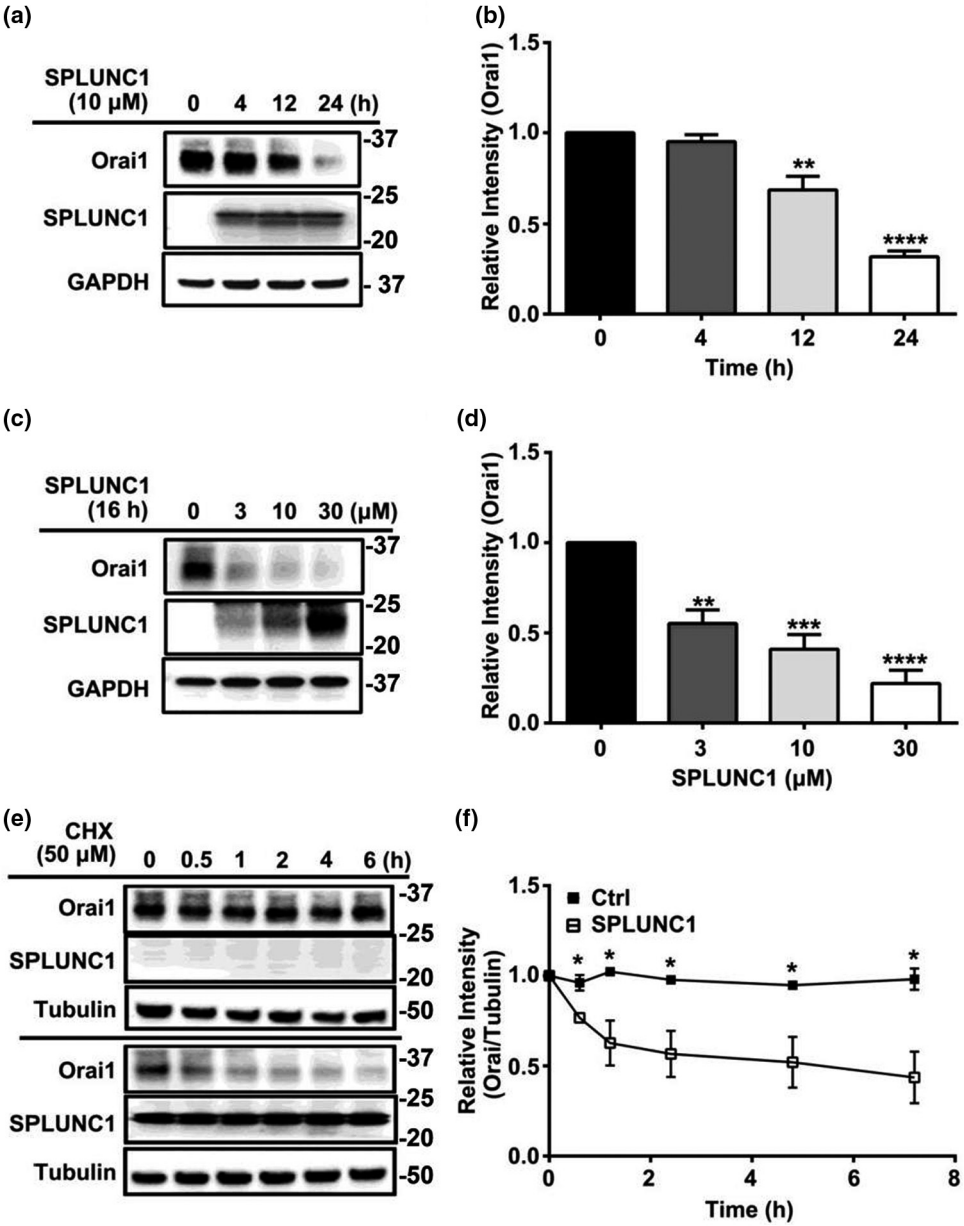
SPLUNC1 decreases Orai1 protein in a time‐ and dose‐dependent fashion. ASM cells were incubated with recombinant SPLUNC1 as indicated. Cell lysates were immunoblotted with an antibody against Orai1 and normalized to GAPDH. (a) Representative immunoblot demonstrating that SPLUNC1 decreases Orai1 protein levels time‐dependently. (b) Orai1 immunoblots from (a) were quantified using Image J and expressed as relative intensity (*n* = 3 experiments). (c) Representative immunoblot demonstrating that SPLUNC1 dose‐dependently decreased Orai1 protein levels. (d) Orai1 immunoblots from (c) were quantified using Image J and expressed as relative intensity (*n* = 4 experiments). (e) Representative immunoblot demonstrating that SPLUNC1 decreases Orai1 protein half‐life in the presence of cycloheximide (CHX) (50 µM). (f) Graph quantifying Orai1 protein levels, expressed as relative intensity change over time (*n* = 3 experiments). *, ** and *** indicate *p* < .05, *p* < .01 and *p* < .001 respectively, compared to each relevant control.

**FIGURE 2:**
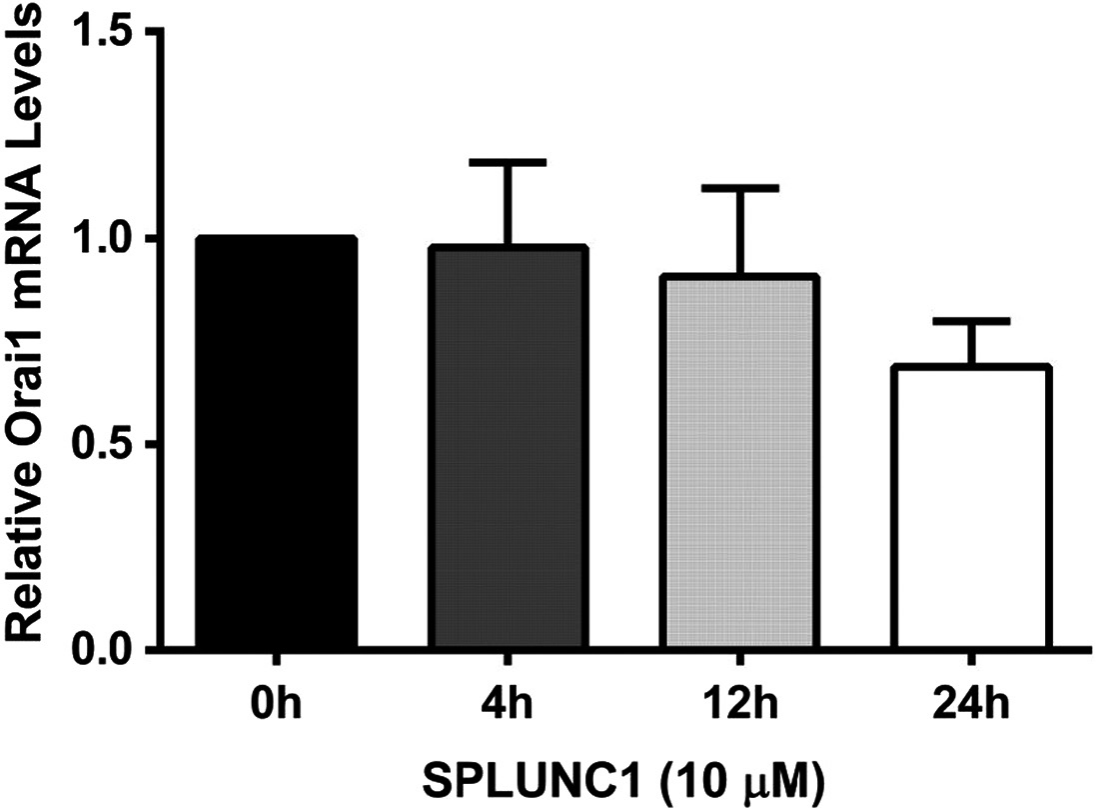
SPLUNC1 does not affect Orai1 mRNA expression. ASM cells were treated with 10 µM SPLUNC1 as indicated, mRNA was extracted and levels of Orai1 mRNA were measured by qRT‐PCR (*n* = 3 experiments). Orai1 expression was normalized to GAPDH.

We previously demonstrated that exogenous SPLUNC1 inhibited Orai1‐mediated Ca^2+^ influx in both human ASM and HEK293T cells, and subsequently identified SPLUNC1 as a binding partner of Orai1 through co‐immunoprecipitation studies (Wu et al., 2017). To better understand this interaction, we performed Western blots on ASM cells exposed to SPLUNC1. 10 µM SPLUNC1 caused a significant reduction in Orai1 protein levels over time (Figure 1A, B). Next, we exposed ASM cells to a range of SPLUNC1 concentrations overnight (16 h). SPLUNC1 decreased Orai1 at all concentrations (Figure 1C, D). To differentiate between reduced synthesis and increased degradation, we inhibited protein synthesis by pretreating cells with 50 µM cycloheximide and chased Orai1 protein degradation in the presence or absence of SPLUNC1. In the presence of cycloheximide, 10 µM SPLUNC1 significantly increased Orai1 degradation (Figure 1E, F). This decrease in Orai1 protein levels was not accompanied by significant changes in mRNA expression (Figure 2), which further indicated that SPLUNC1 decreases Orai1 levels at the protein rather than the transcript level.

### 2.2 SPLUNC1 induces allostery in Orai1

**FIGURE 3:**
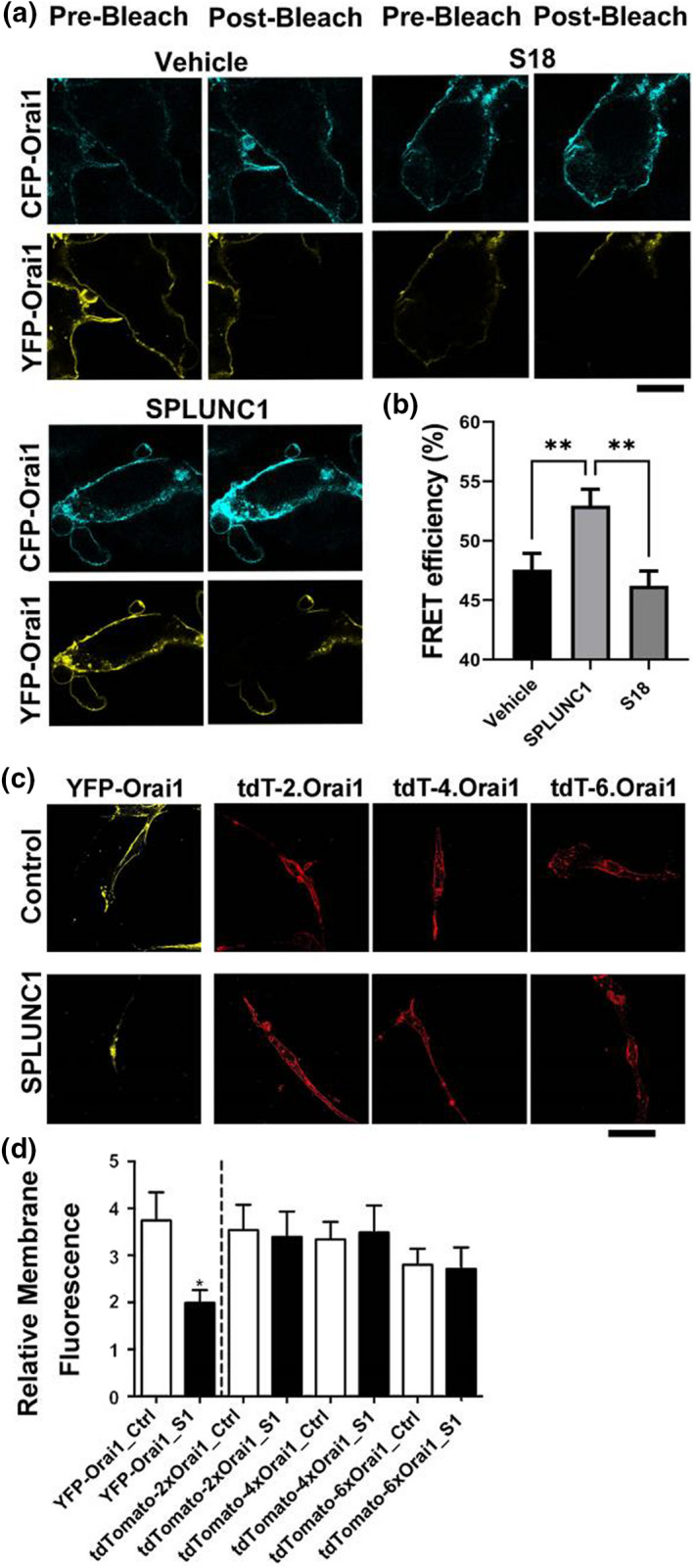
SPLUNC1‐induced allosteric changes in Orai1 are required for Orai1 internalization. (a) Representative confocal images of HEK293T cells before and after acceptor photobleaching between YPF‐Orai1 and CPF‐Orai1. (b) Change in Orai1‐Orai1 FRET efficiencies in vehicle, SPLUNC1, and S18‐ treated groups as indicated (*n* = 12–20 cells/treatment group from 3 separate experiments). (c) Representative images of ASM cells expressing covalently‐linked Orai1 multimers ± SPLUNC1. All three Orai1 polymers were labelled with TdTomato. YFP‐Orai1 monomers served as controls. 24 h post‐transfection, cells were incubated with or without 10 µM SPLUNC1 for 4 h before fixation. (d) Bar graph showing fluorescence intensity of membrane Orai1 constructs before and after SPLUNC1 treatment as indicated (*n* = 4–6 coverslips/group). * and ** indicate *p* < .05 and *p* < .01 respectively. Scale bars are 10 µM.

Since SPLUNC1 promoted Orai1 degradation (Figure 1) and SPLUNC1 does not bind to ASM cells unless Orai1 is expressed (Wu et al., 2017), we reasoned that SPLUNC1 was an allosteric regulator of Orai1 that upon binding extracellularly, would cause a conformational change in Orai1. To test for this, we co‐expressed CFP‐Orai1 and YFP‐Orai1 into HEK293T cells and measured plasma membrane Fӧrster Resonance Energy Transfer (FRET) as described (Kim et al., 2018). Extracellular SPLUNC1 binding significantly increased plasma membrane and/or near plasma membrane FRET between CFP‐Orai1 and YFP‐Orai1. S18, a peptide corresponding to the N‐terminus of SPLUNC1, that does not interact with Orai1 (Wu et al., 2017), had no effect on FRET efficiency (Figure 3A, B). Since these data indicated that SPLUNC1 acted allosterically, we next expressed Orai1 concatemers, where 2, 4, or 6 Orai1 cDNAs were physically linked to form a functional channel (Cai et al., 2016). The 2x, 4x, and 6x Orai1 concatemers were transfected into ASM cells, and confocal microscopy was performed to determine plasma membrane Orai1 levels after exposure to vehicle or SPLUNC1. In the concatemers, membrane fluorescence intensities of Orai1 were unaffected by SPLUNC1 exposure. (Figure 3C, D). This effect was not cell‐type dependent, and we observed an identical effect when these constructs were expressed in HEK293T cells (Figure S1).

### 2.3 SPLUNC1 induces Nedd4‐2‐mediated ubiquitination of Orai1

**FIGURE 4:**
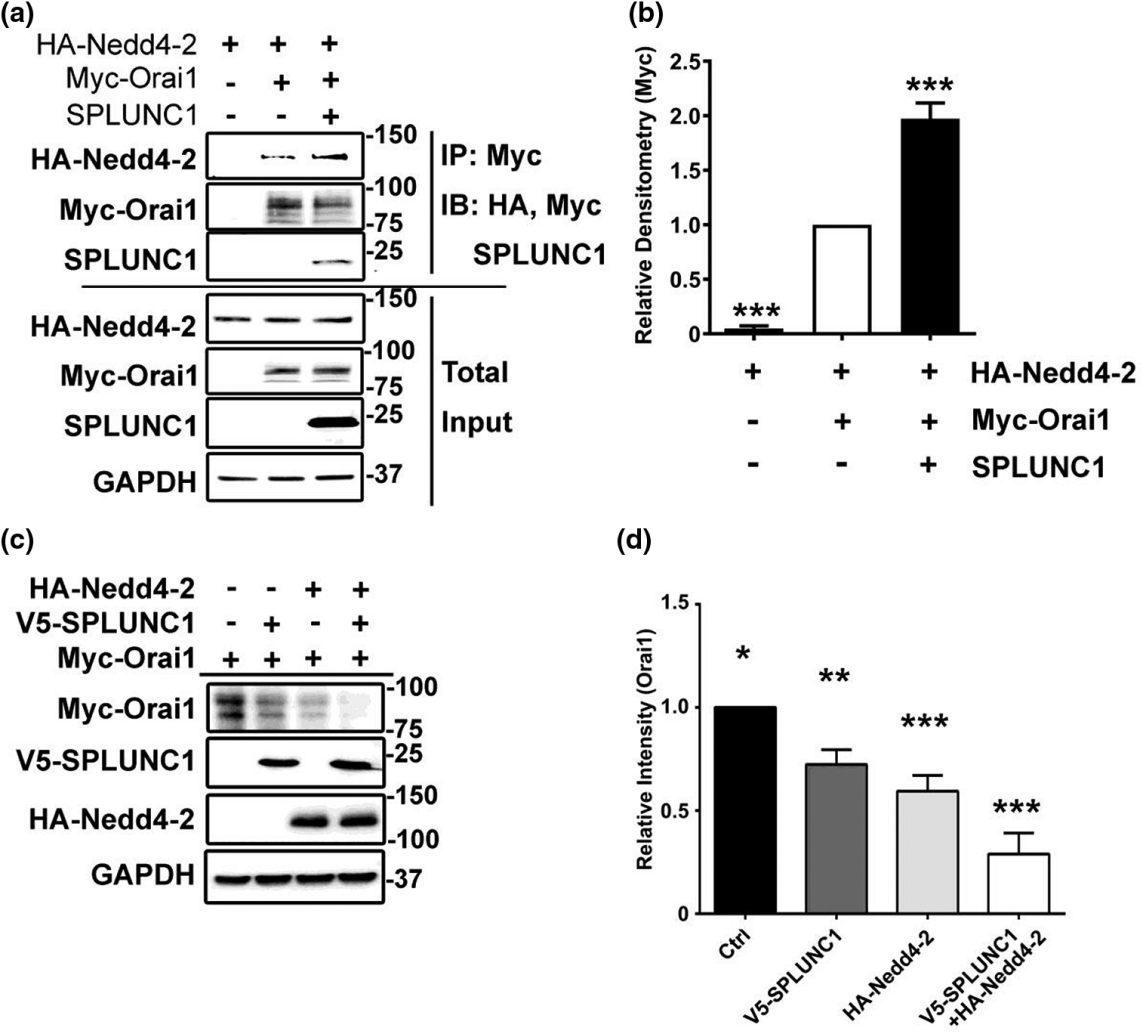
Nedd4‐2 co‐immunoprecipitates with Orai1. (a) HEK293T cells were co‐transfected with Myc‐Orai1 ± V5‐SPLUNC1 and ± HA‐Nedd4.2. Cell lysates from HEK293T cells co‐transfected with Myc‐Orai1 and HA‐Nedd4‐2 in the presence or absence of SPLUNC1 was used for immunoprecipitation analysis. HA‐Nedd4‐2 co‐immunoprecipitated with Myc‐Orai1; SPLUNC1 increased this interaction. Data represent three independent experiments. (b) Intensity of HA immunoblots, detecting HA‐Nedd4‐2, from (a), were quantified and expressed as relative intensity (*n* = 3 experiments). (c) Representative immunoblot demonstrating that SPLUNC1 and Nedd4‐2 decrease Orai1 protein levels in HEK293T cells. (d) Orai1 immunoblots from (C; *n* = 3 experiments). *, ** and *** indicate *p* < .05, *p* < .01 and *p* < .001 respectively.

The ubiquitin ligase Nedd4‐2 targets a wide variety of membrane proteins for lysosomal degradation (Laedermann et al., 2013; Zhou et al., 2007), and SPLUNC1 has previously been shown to ubiquitinate other ion channels by increasing their interactions with Nedd4‐2 (Kim et al., 2018). We, therefore, tested the hypothesis that Orai1 was ubiquitinated by Nedd4‐2. We transfected HA‐tagged Nedd4‐2 ± myc‐tagged Orai1 ± V5‐tagged SPLUNC1 and probed for Myc. We found that HA‐Nedd4‐2 and SPLUNC1 could be co‐immunoprecipitated and that SPLUNC1 increased this interaction (Figure 4A, B). Consistent with Nedd4‐2 being required for Orai1 degradation, overexpression of Nedd4‐2 or SPLUNC1 alone significantly reduced Orai1 protein levels, and co‐transfection of Nedd4‐2 and SPLUNC1 further decreased Orai1 to ~50% of levels seen in control cells (Figure 4A, D). Taken together, these data indicated that SPLUNC1 and Nedd4‐2 had a synergistic effect on Orai1 protein degradation.

**FIGURE 5:**
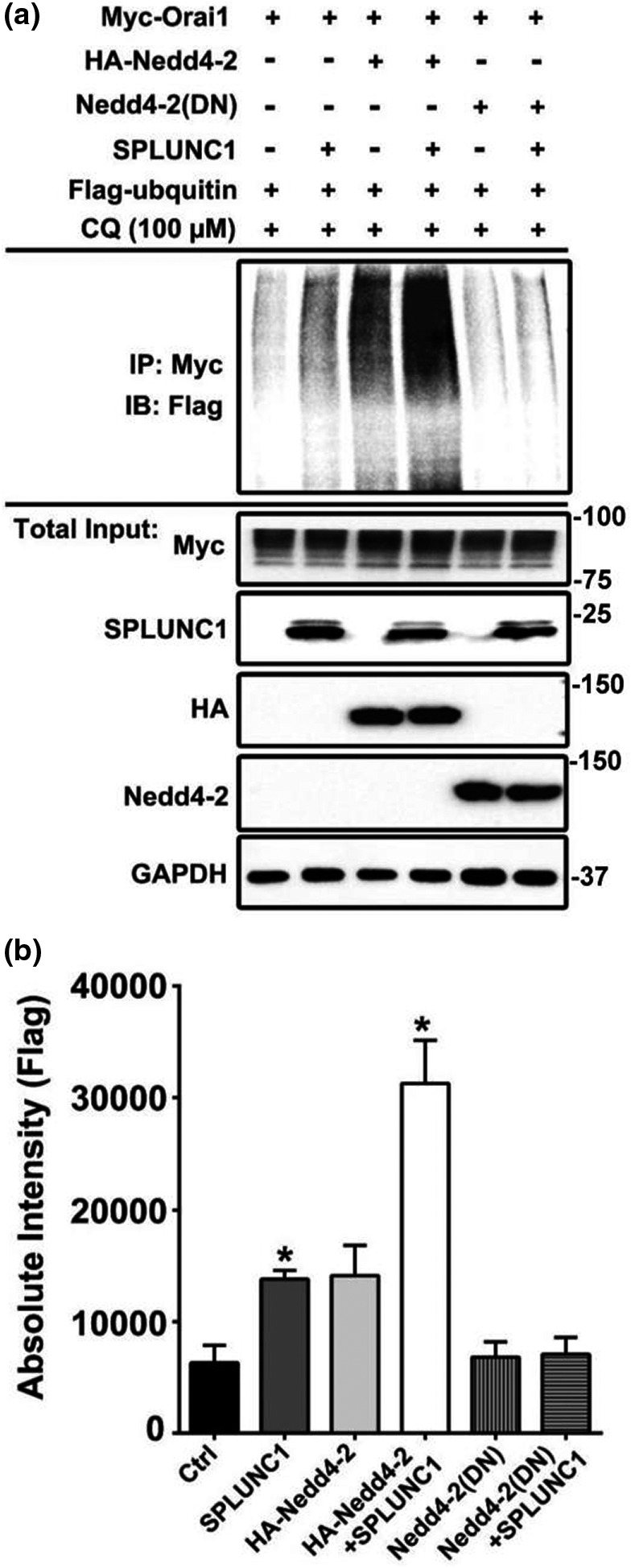
Nedd4‐2 is necessary for SPLUNC1‐mediated Orai1 ubiquitination. ASM cells were transfected with the indicated constructs and incubated ± recombinant SPLUNC1 for 16 h in the presence of 100 µM CQ to prevent SPLUNC1 degradation. (a) Ubiquitinated Orai1 was detected by immunoprecipitation using an anti‐myc epitope antibody recognizing myc‐tagged Orai1, followed by immunoblotting with anti‐FLAG for detecting Flag‐ubiquitin. Representative immunoblot showing that functional Nedd4‐2 is necessary for SPLUNC1‐mediated Orai1 ubiquitination. (b) Anti‐Flag immunoblots from (a) were quantified using Image J and expressed as absolute intensity (*n* = 4 experiments). * and ** indicate *p* < .05 and *p* < .001 different to control respectively. # indicates *p* < .05 different to SPLUNC1.

Next, we measured the ubiquitination of Orai1 in the presence of SPLUNC1 and/or Nedd4‐2. Consistent with a previous study (Eylenstein et al., 2011), overexpression of Nedd4‐2 alone was sufficient to increase Orai1 ubiquitination (Figure 5A, B). Similarly, SPLUNC1 overexpression also promoted Orai1 ubiquitination, and co‐expression of both SPLUNC1 and Nedd4‐2 synergistically increased Orai1 ubiquitination (Figure 5A, B). To test whether the functional integrity of Nedd4‐2 was necessary for SPLUNC1‐mediated Orai1 ubiquitination, we transfected HEK293T cells with wild‐type Nedd4‐2, or the catalytically inert Nedd4‐2^C812A^ ± SPLUNC1 (Zhou & Snyder, 2005). Again, wild‐type Nedd4‐2 and SPLUNC1 synergistically increased Orai1 ubiquitination levels (Figure 5A, B). However, this effect was abolished by overexpression of Nedd4‐2^C812A^, and in the presence of this construct, Orai1 ubiquitination could not be rescued by the addition of SPLUNC1 (Figure 5A, B).

### 2.4 SPLUNC1 induces lysosomal degradation of Orai1

**FIGURE 6:**
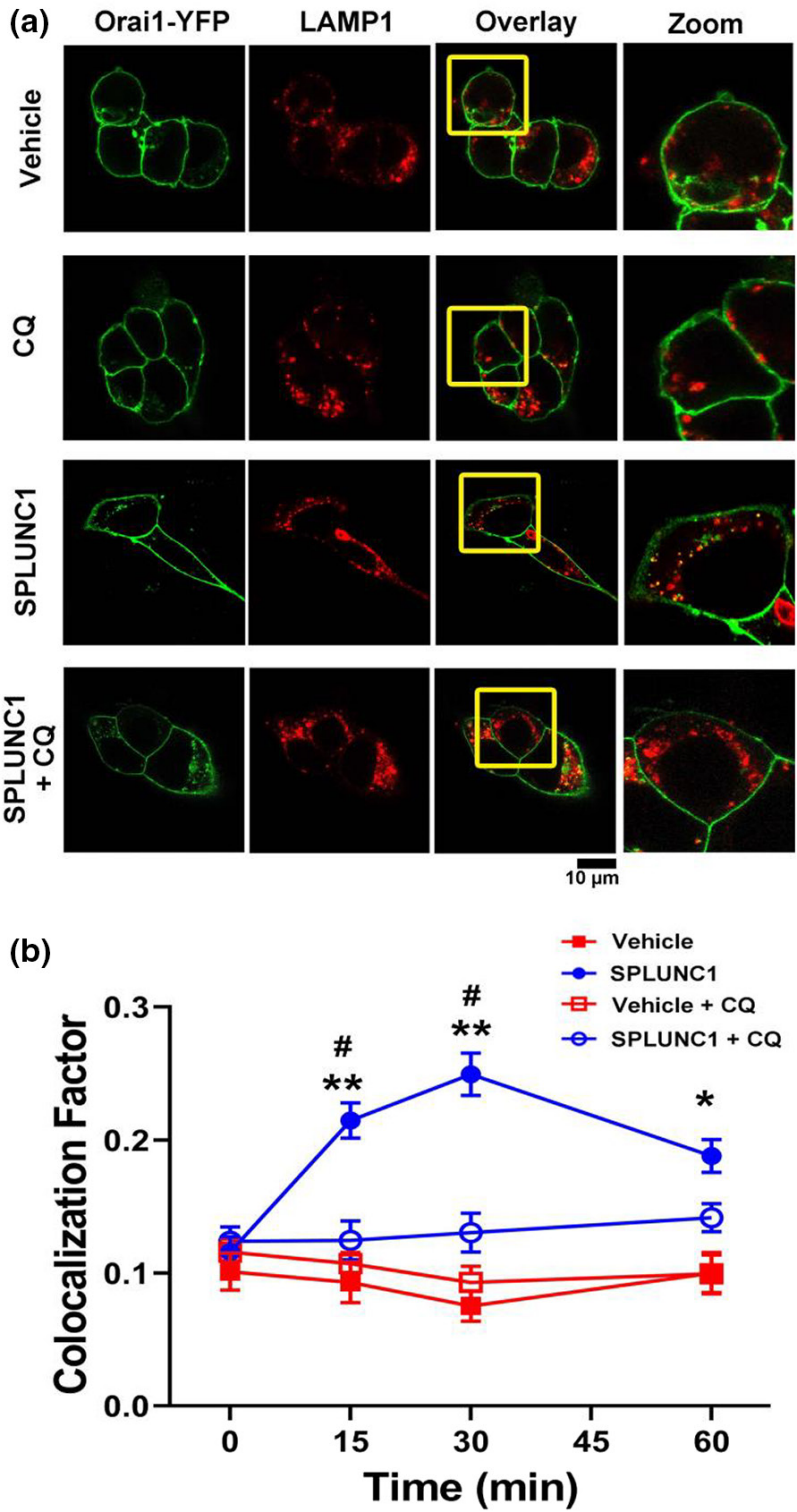
SPLUNC1 promotes Orai1 internalization to lysosomes. HEK293T cells were treated with chloroquine or vehicle and fixed at 0–60 min in paraformaldehyde. Cells were stained with antibodies raised against LAMP‐1 and the relevant secondary antibody and them imaged by confocal microscopy. (a) XY‐confocal micrographs showing Orai1 (green) and LAMP1 (red) 30 min after addition of 10 µM SPLUNC1. In some cases, cells were pretreated for 1 h with 100 µM chloroquine (CQ). (b) Graph showing change in colocalization between Orai1‐YFP and LAMP‐1 over time (*n* = 12–57 cells from four independent experiments per data point). * and ** indicates *p* < .05 and *p* < .01 compared to control. # indicates *p* < .01 different to vehicle at each time point.

In mammalian cells, the two major routes for protein degradation are via the proteasome or the lysosome, which are sensitive to Mg132 and chloroquine respectively (Lee & Goldberg, 1998; Tanaka et al., 1986). Previous studies have found that Orai1 is sensitive to lysosomal but not proteasomal inhibitors (Lee et al., 2013). Thus, we hypothesized that Orai1 would be trafficked to the lysosomes for degradation. To determine Orai1’s fate after SPLUNC1‐induced internalization and ubiquitination, we looked for co‐localization between Orai1‐YFP and LAMP1 in HEK293T cells after SPLUNC1 exposure. Cultures were fixed at timed intervals after SPLUNC1 or vehicle addition, antibody stained and imaged by confocal microscopy. Little co‐localization was seen between Orai1 and LAMP1 after vehicle addition. In contrast, SPLUNC1 caused a significant increase in co‐localization between Orai1 and LAMP1 within 15 min, which persisted for the duration of the experiment (Figure 6A, B). Pretreatment with chloroquine (100 µM) had no effect on basal Orai1‐LAMP1 co‐localization, but abolished the SPLUNC1‐induced co‐localization (Figure 6A, B).

## 3 DISCUSSION

We have previously reported that SPLUNC1 inhibits SOCE by binding to and internalizing Orai1 (Wu et al., 2017), and we speculate that the lack of this interaction caused the spontaneous airway hyperreactivity in SPLUNC1^−/−^ mice. SPLUNC1 did not bind to ASM cells unless Orai1 was present, suggesting that SPLUNC1 and Orai1 directly interacted. However, the mechanism of inhibition/internalization was poorly understood. Here, we have demonstrated that SPLUNC1 is an allosteric regulator of Orai1 that targets Orai1 for ubiquitination and degradation in the lysosome.

### 3.1 SPLUNC1 as an allosteric regulator of Orai1

Our data indicated that the addition of extracellular SPLUNC1 reduced Orai1 protein but not transcript levels (Figures 1 and 2). However, to the best of our knowledge, the majority, if not all biological mechanisms that regulate ion channel density are intracellular, suggesting that SPLUNC1 is an allosteric regulator of Orai1 that binds extracellularly and causes intracellular conformational changes in Orai1 that lead to its degradation. To test for this, we expressed Orai1 constructs with C‐terminally‐linked, cytoplasmic CFP and YFP. We then measured CFP and YFP FRET using the acceptor photobleaching method as previously described (Kim et al., 2018). Indeed, using this approach, we found that extracellular SPLUNC1 induced significant changes in intracellular Orai1 FRET whilst Orai1 was still in the vicinity of the plasma (Figure 3A, B), thus indicating that SPLUNC1 induced allosteric changes in Orai1. To further confirm that SPLUNC1 induced conformational changes in Orai1, we expressed a series of Orai1 concatemers (Cai et al., 2016). Since these concatemers were physically constrained, we hypothesized that they would not be able to internalize. Indeed, our data indicated that, unlike the monomeric Orai1, neither the 2x, 4x, nor 6x concatemers were internalized by SPLUNC1 (Figure 3C, D, S1). The Orai1 crystal structure indicates that Orai1 is hexameric (Hou et al., 2012), and the Orai1 6x concatemers have previously been shown to have biophysical characteristics indistinguishable from native Orai1 channels, including store‐dependent activation and Ca^2+^‐dependent inactivation (Yen et al., 2016). Thus, prior to SPLUNC1 exposure, it is likely that these concatemers were physiologically functional. Orai1 has previously been shown to be an allosteric regulator of the Ca^2+^‐sensitive protein, calmodulin (Maganti et al., 2019). However, to the best of our knowledge, this is the first demonstration of an extracellular regulatory protein that can confer allostery in Orai1. Orai1 is regulated by SPLUNC1’s C‐terminal α6 region (DITLVHDIVNMLIHGL) (Wrennall et al., 2022; Wu et al., 2017). We previously found that SPLUNC1’s N‐terminal S18 region (GGLPVPLDQTLPLNVNPA) was an allosteric regulator of ENaC (Kim et al., 2018). Indeed, SPLUNC1 altered FRET between all three ENaC subunits and internalized ENaC. However, despite being from the same protein as α6, the S18 peptide did not inhibit SOCE (Wu et al., 2017). Since two peptides from SPLUNC1 can regulate different ion channels, it is tempting to speculate that additional proteins beyond SPLUNC1 can also bind to and regulate ion channels in the lung lumen and on other mucosal surfaces. However, additional studies will be required to test this.

### 3.2 Trafficking to lysosomes

SPLUNC1 significantly lowered total Orai1 protein but not mRNA levels over time (Figures 1, 2, 5). SPLUNC1’s effects on Orai1 persisted for 24 h (Figure 1), suggesting that SPLUNC1 can modify total Orai1 levels. The lysosomal deacidifier chloroquine significantly increased co‐localization between Orai1 and the lysosomal marker protein LAMP1 (Figure 6). This is consistent with previous reports that Orai1 is sensitive to lysosomal inhibitors (Lee et al., 2013), and suggested that SPLUNC1 directs Orai1 to the lysosome. We have not explored what would happen to Orai1 after SPLUNC1 has been removed, but we posit that any subsequent increase in Orai1 would be due to de novo Orai1 synthesis rather than recycling of internalized Orai1 to the plasma membrane. Interestingly, during meiosis, Orai1 is endocytosed, but does not go to lysosomes (Yu et al., 2010). In contrast, ubiquillin 1 also increases Orai1 ubiquitination and sends it to lysosomes for degradation (Lee et al., 2013), suggesting that Orai1’s intracellular fate may be variable depending on the regulatory process and physiological needs of the cell. However, further studies are needed to fully understand Orai1 synthesis/recycling in the context of changing SPLUNC1 levels.

### 3.3 SPLUNC1’s Physiological Role and Clinical Implications

SPLUNC1 is highly abundant in the airway surface liquid that lines the lungs, suggesting SPLUNC1’s regulation of Orai1 is physiologically relevant. Under normal conditions, SPLUNC1 is thought to be predominantly expressed in the oral, nasopharyngeal, and large airways, and is poorly expressed in the small airways (Bingle & Craven, 2002; Britto et al., 2013). The upper and large airways are much more exposed to inhaled pathogens than the small airways and alveolar regions. Indeed, this region of the airways is potentially exposed to inhaled pathogens, pollution, and/or allergens with every breath. Thus, SPLUNC1 may act as a brake on Orai1 activity in the upper/large airways to prevent excessive inflammation. Interestingly, SPLUNC1 is highly susceptible to degradation by neutrophil elastase (Jiang et al., 2013) and SPLUNC1 levels are significantly reduced in the cystic fibrosis (CF) lung, which typically has chronically elevated neutrophil elastase levels (Webster et al., 2018). Moreover, SPLUNC1 levels are also significantly diminished in asthmatic sputum, although in asthma patients, SPLUNC1 is downregulated at the gene level, rather than due to protease mediated degradation (Wu et al., 2017). Thus, we speculate that the lack of SPLUNC1 may contribute to the hyperinflammation seen in CF and asthmatic lungs. Indeed, SPLUNC1 acts as a gene modifier in both CF and asthma (Saferali et al., 2015; Schaefer et al., 2019) and we speculate that reduced SPLUNC1 levels may aberrantly increase Orai1 levels and SOCE in both diseases, leading to increased inflammation. Thus, the addition of recombinant SPLUNC1, or a SPLUNC1 analog may serve as a novel therapy to reduce lung Orai1 levels and reduce inflammation. Indeed, we have recently shown that a peptidomimetic of SPLUNC1’s α6 region ameliorates inflammation in a house dust mite‐sensitized mouse model of asthma (Wrennall et al., 2022), suggesting that SPLUNC1 replacement therapy may be a novel approach to treat pulmonary inflammation.

### 3.4 Conclusions

We have found that SPLUNC1 is an allosteric modulator of Orai1 that promotes Orai1 ubiquitination and lysosomal degradation. To the best of our knowledge, this is the first demonstration that an extracellular protein can regulate Orai1 protein levels and the first demonstration of an allosteric regulator of Orai1. While SPLUNC1 is thought to be predominantly expressed in the pulmonary system and oral cavity, whether SPLUNC1, or other, as‐yet‐unidentified proteins can similarly promote Orai1 ubiquitination in other tissues remains to be determined.

## 4 COMPETING FINANCIAL INTERESTS

M. Flori Sassano, Saira Ahmad, and Robert Tarran have equity in Eldec Pharmaceuticals Inc. The other authors declare no competing financial interests.

## AUTHOR CONTRIBUTIONS

Tongde Wu, Alexandra S. Goriounova, Erin N. Worthington, Joe A. Wrennall, and Arunava Ghosh conducted experiments. Tongde Wu, Alexandra S. Goriounova, Erin N. Worthington, Joe A. Wrennall, and R. Tarran analyzed the data. Tongde Wu, Erin N. Worthington, and Robert Tarran designed experiments. Alexandra S. Goriounova, Saira Ahmad, M. Flori Sassano, and Robert Tarran wrote the manuscript. All other authors edited and approved the manuscript.

## Supporting information



Fig S1
