# Supplementary material for: SPLUNC1 is a negative regulator of the Orai1 Ca2+ channel
Source: Physiol Rep. 2022 May 17;10(10):e15306. doi: 10.14814/phy2.15306 (PMC9114653; doi:10.14814/phy2.15306)
Supplement: Supplementary file 1 — Fig S1 [file PHY2-10-e15306-s001.docx]

**Supplemental data for:**

**SPLUNC1 is a Negative Regulator of the Orai1 Ca^2+^ Channel**

**Tongde Wu^1^, Alexandra S. Goriounova^2^, Erin N. Worthington^3#^, Joe A. Wrennall^1^, Arunava Ghosh^1^, Saira Ahmad^1^, M. Flori Sassano^1^ and Robert Tarran^1*^**

**Affiliations:** ^1^Department of Cell Biology & Physiology, ^2^Department of Pharmacology, ^3^Divison of Pulmonology, The University of North Carolina at Chapel Hill, NC, 27599, USA.

**#**Current address: Department of Pediatric Pulmonology, Virginia Tech Carilion School of Medicine, Roanoke, VA 24016

*Correspondence to: Robert Tarran, PhD

Address: Department of Cell Biology & Physiology

5109C Neuroscience Research Building

115 Mason Farm Road,

University of North Carolina at Chapel Hill,

Chapel Hill, NC, 27599, USA.

Email: robert_tarran@med.unc.edu

Phone: 919-966-7052

**Keywords:** Airway smooth muscle; calcium; NEDD4-2; BPIFA1; FRET

**Author Contributions:** T. Wu, A.S. Goriounova, E.N. Worthington, J.A. Wrennall, and A. Ghosh conducted experiments. T. Wu, A.S. Goriounova, E.N. Worthington, J.A. Wrennall, and R. Tarran analyzed data. T. Wu, E.N. Worthington, and R. Tarran designed experiments. A.S. Goriounova, S. Ahmad, M.F. Sassano, and R. Tarran wrote the manuscript. All other authors edited and approved the manuscript.


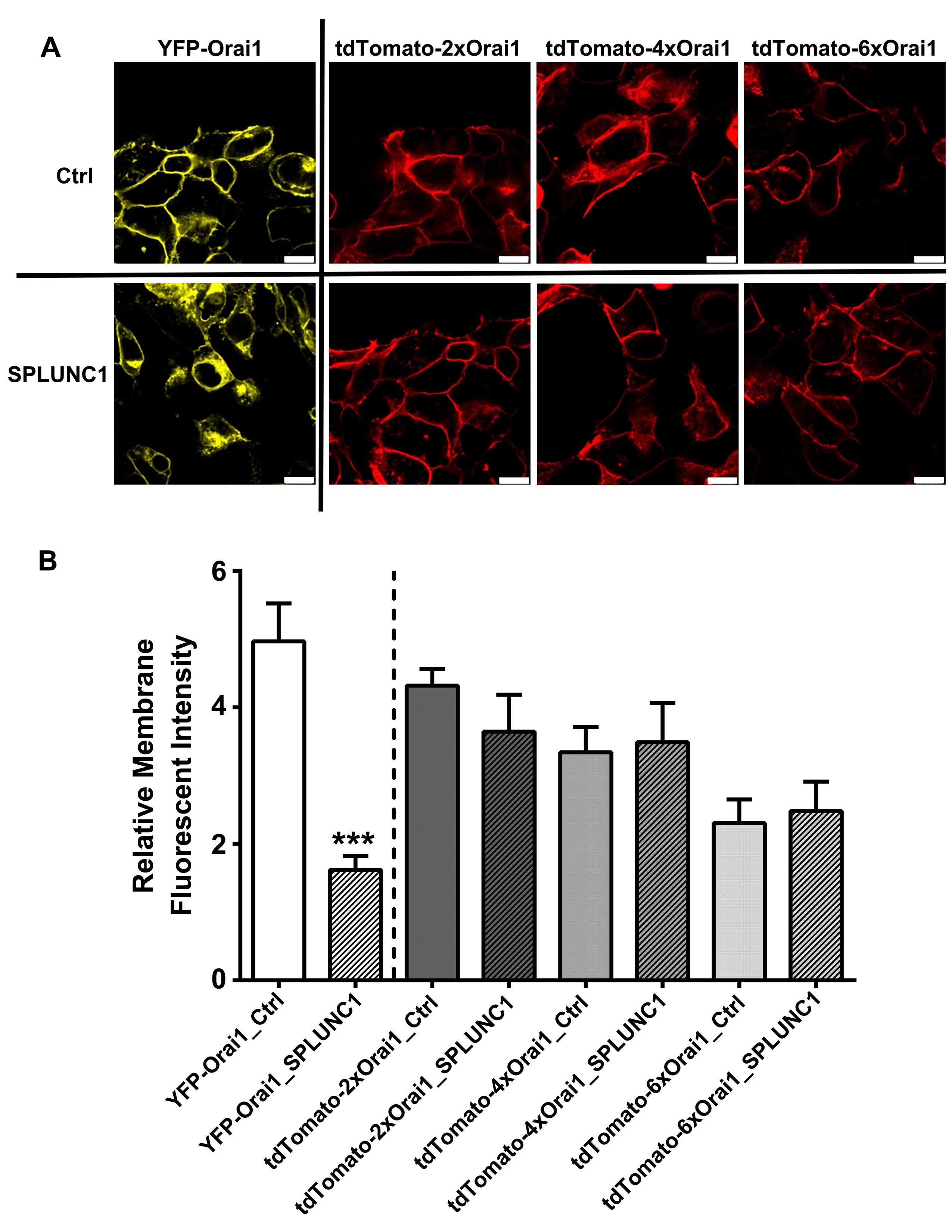


**Supplementary Figure 1.** **SPLUNC1 does not internalize covalently linked Orai1 polymers in HEK293T cells. (A)** The cellular localization of covalently linked Orai1 polymers in the presence or absence of SPLUNC1 was detected by confocal microscopy. All three Orai1 polymers are tdTomato tagged. YFP-Orai1 was served as control. 24 h post-transfection, cells were incubated with or without 10 μM SPLUNC1 for 4 h before fixation. **(B)** Fluorescence intensity of membrane-retained Orai1 constructs was quantified using Image J and expressed as relative membrane intensity both before and after SPLUNC1 treatment (n = 4-6 coverslips/group). The data were analyzed using Mann-Whitney test. *** indicates P<0.001 compared to control. Scale bars are 10 µm.
